# Supplementary material for: Ca2+–Calmodulin Dependent Wound Repair in Dictyostelium Cell Membrane
Source: Cells. 2020 Apr 23;9(4):1058. doi: 10.3390/cells9041058 (PMC7226253; doi:10.3390/cells9041058)
Supplement: Supplementary file 1 [file cells-09-01058-s001.pdf]

cells

MDPI

Article

Ca²⁺-Calmodulin Dependent Wound Repair in *Dictyostelium* Cell Membrane

Md. Shahabe Uddin Talukder [1,2], Mst. Shaela Pervin [1,3], Md. Istiaq Obaidi Tanvir [1], Koushiro Fujimoto [1], Masahito Tanaka [1], Go Itoh[4], and Shigehiko Yumura [1*]

[1] Graduate School of Sciences and Technology for Innovation, Yamaguchi University, Yamaguchi 753-8511, Japan; shahabeuddin@yahoo.com (M.S.U.T.); sprvn@yahoo.com (M.S.P.); tanviristiaq@gmail.com (M.I.O.T.); i501wz@yamaguchi-u.ac.jp (K.F.); g003wf@yamaguchi-u.ac.jp (M.T.); yumura@yamaguchi-u.ac.jp (S.Y.)

[2] Institute of Food and Radiation Biology, AERE, Bangladesh Atomic Energy Commission, Bangladesh.

[3] Rajshahi Diabetic Association General Hospital, Luxmipur, Jhautala, Rajshahi-6000, Bangladesh.

[4] Department of Molecular Medicine and Biochemistry, Akita University Graduate School of Medicine, Akita, 010-8543, Japan; goitoh@med.akita-u.ac.jp (G.I.)

* Correspondence: yumura@yamaguchi-u.ac.jp (S.Y.); Tel.: +81-83-933-5717; Fax: +81-83-933-5717

Supplemental Data

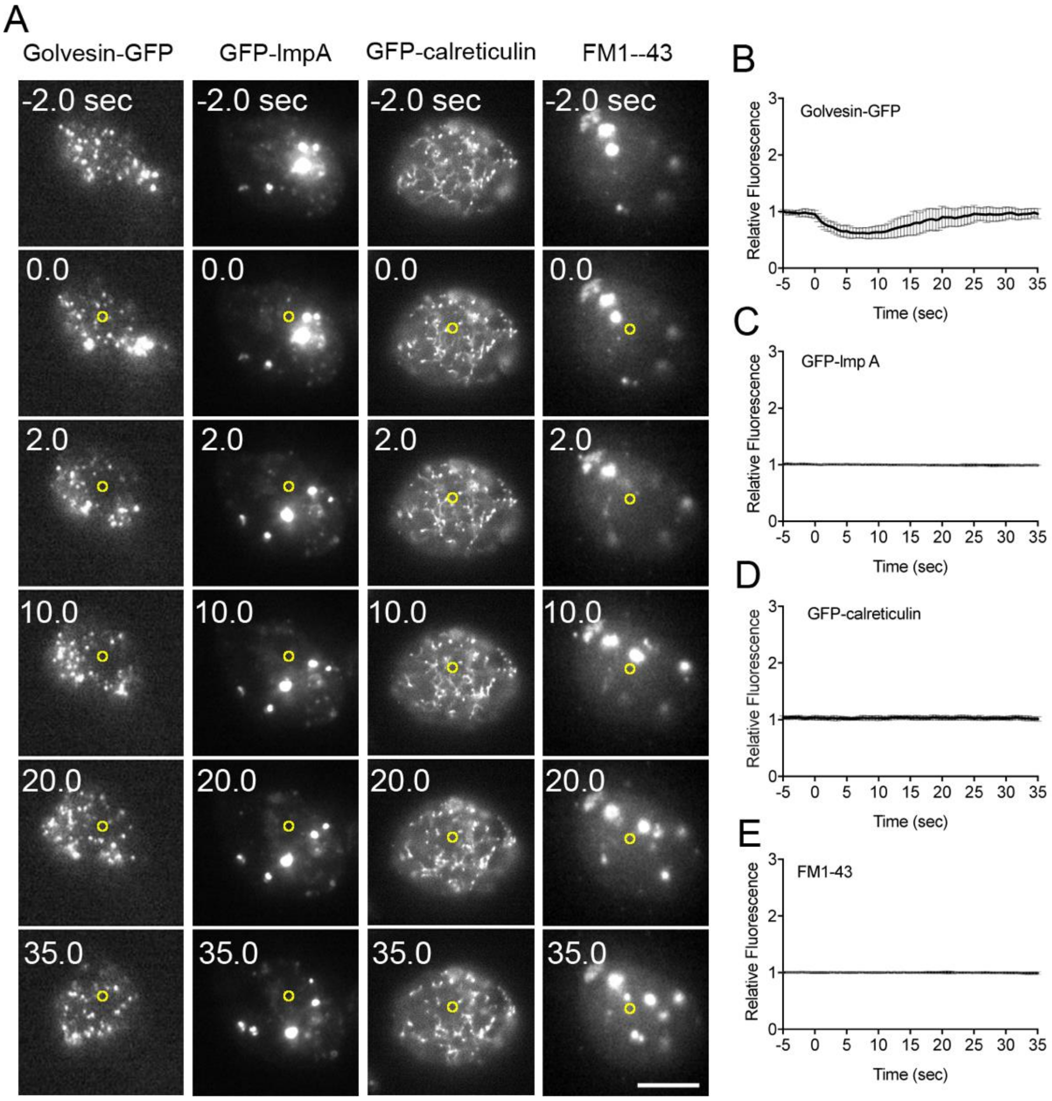

Supplemental Figure S1. Golgi-derived vesicles, lysosome, endoplasmic reticulum, and recycling endosomes did not accumulate at the wound site. (A) Typical fluorescence images under TIRF microscopy when cells expressing golvesin-GFP, GFP-lmpA, or GFP-calreticulin, and FM dye-stained cells were wounded by laserporation (yellow circles). To visualize the recycling endosomes, 30 min after the cells were incubated with FM1-43, they were washed by media exchange. Bars, 10 μm. (B-E) Time courses of fluorescence intensity of each probe at wound site (n = 25, each). Note that golvesin-GFP transiently disappeared from the wound site after wounding.
